# Importin α3/Qip1 Is Involved in Multiplication of Mutant Influenza Virus with Alanine Mutation at Amino Acid 9 Independently of Nuclear Transport Function

**DOI:** 10.1371/journal.pone.0055765

**Published:** 2013-01-30

**Authors:** Yutaka Sasaki, Kyoji Hagiwara, Michinori Kakisaka, Kazunori Yamada, Tomoyuki Murakami, Yoko Aida

**Affiliations:** 1 Viral Infectious Diseases Unit, RIKEN, Wako, Saitama, Japan; 2 Laboratory of Viral Infectious Diseases, Department of Medical Genome Sciences, Graduate School of Frontier Science, The University of Tokyo, Wako, Saitama, Japan; St. Georges University of London, United Kingdom

## Abstract

The nucleoprotein (NP) of influenza A virus is transported into the nucleus via the classical importin α/β pathway, and proceeds via nuclear localization signals (NLSs) recognized by importin α molecules. Although NP binds to importin α isoforms Rch1, Qip1 and NPI-1, the role of each individual isoform during the nuclear transport of NP and replication of the influenza virus remains unknown. In this study, we examined the contribution of importin α isoforms for nuclear localization of NP and viral growth using a panel of NP mutants containing serial alanine replacements within an unconventional NLS of NP. Alanine mutation at amino acid 8 (R8A) caused a significant reduction in the nuclear localization and binding to the three importin isoforms. The R8A NP mutant virus did not generate by reverse-genetics approach. This indicates that position 8 is the main site that mediates nuclear localization via interactions with Rch1, Qip1 and NPI-1, and subsequent viral production. This was confirmed by the finding that the conservation of amino acid 8 in human- and avian-origin influenza virus NP was necessary for virus propagation. By contrast, another mutant, S9A NP, which localized in the nucleus, caused a reduction in viral growth and vRNA transcription, suggesting that the unconventional NLS within NP may be associated with nuclear transport, vRNA transcription and viral replication through independent pathways. Interestingly, the N-terminal 110-amino acid region, which contained the unconventional NLS with S9A mutation, mainly bound to Qip1. Furthermore, activities of vRNA transcription and replication of S9A NP mutants were decreased by silencing Qip1 in without changing nuclear localization, indicating that Qip1 involves in multiplication of S9A mutant virus independently of nuclear transport function. Collectively, our results demonstrate the unconventional NLS within NP might have the additional ability to regulate the viral replication that is independent of nuclear localization activity via interactions with Qip1.

## Introduction

The influenza A virus is a negative-stranded RNA virus with eight genome segments surrounded by a layer of matrix (M1) proteins and a lipid bilayer derived from the host cell [1], [2], [3]. The viral RNA (vRNA) is associated with three polymerase subunit proteins (PB2, PB1 and PA) and the viral nucleoprotein (NP), which is composed of viral ribonucleoprotein complexes (vRNPs) [4]. After adsorption to host cells and internalization via endocytosis, viruses are incorporated into endosomes within the cells. The vRNPs are released into the cytoplasm by a pH-dependent fusion of hemagglutinin to the endosomal membranes [5], [6]. The vRNPs are then transported into the nucleus through the nuclear pores. Unlike most other RNA viruses, influenza vRNAs are transcribed and replicated within the host cell nucleus via their polymerase subunits [7], [8], [9]. After export into the cytoplasm, vRNPs are assembled under the plasma membrane and incorporated into viral particles [10]. The transport of vRNPs into the nucleus takes place through the classical nuclear import pathway, which relies on importin α/β transport systems. The adaptor molecule, importin α, comprises a flexible N-terminal importin β-binding (IBB) domain and a highly structured domain comprising 10 tandem armadillo (ARM) repeats, and recognizes the cargo borne by the nuclear localization signal (NLS). Importin β docks the complex to the nuclear pores and translocates it to the nucleus [11], [12]. Thus, the importin α/β transport system facilitates the transport of influenza virus vRNPs into the nucleus [11], [12].

The importin α gene family has undergone considerable expansion during the course of eukaryotic evolution. Human importin α is a helical molecule with at least seven isoforms [13], [14], [15], which are categorized into three phylogenetically distinct subfamilies (α1, α2 and α3) based on their amino acid sequence similarity. Subfamily α1 includes importin α5 (NPI-1), importin α6 (KPNA5), and importin α7 (KPNA6). Subfamily α2 includes importin α1 (Rch1) and the recently-reported importin α8 (KPNA7) [14], [15]. Subfamily α3 includes importin α3 (Qip1) and importin α4 (KPNA3). These subfamilies are 50% homologous [13]. Many studies show that the importin α subfamilies differ in terms of their efficiency with respect to classical substrate-specific import and show unique expression patterns in various tissues and cells, and also that their functions depend on the state of cellular metabolism and differentiation [16], [17], [18], [19], [20].

The component proteins of vRNPs, PB2, PB1, PA and NP contain an NLS within their sequences [21], [22], [23], [24]. PB2 is independently transported to the nucleus, whereas PB1 and PA form a dimer in the cytoplasm, which is then transported into the nucleus [25]. NPs are major components of vRNPs, which are bound to vRNA at a distance of 24 nucleotides [26], [27]. NP contains at least three NLSs: one at amino acids 3–13 (the unconventional NLS), one at amino acids 90–121 (the overlapping bipartite NLS), and one at amino acids 198–216 (the bipartite NLS) [24], [28], [29]. Of these, the unconventional NLS is indispensable for the nuclear transport of NP and vRNP [11], [12], [24]. Fluorescence microscope images and experimental infection of cells with alanine-substituted mutants of the unconventional NLS show that two amino acids (at positions 7 and 8) are essential for the nuclear localization of NP, although their contribution to viral replication and viral mRNA transcription is limited [30], [31]. Interestingly, NP binds to Rch1, Qip1 and NPI-1 (all isoforms of importin α) [24]. NP binds to the C-terminal NLS binding site of NPI-1 and Qip1, which comprise ARM repeats 7–9 in NPI-1 and ARM repeat 8 in Qip1 [18]; however, the exact regions of NP that are involved in binding Rch1, Qip1 and NPI-1 are unknown. Furthermore, the importin α family, which also includes importin α7, is associated not only with nuclear transport of NP, but also with cell tropism, and host adaptation and replication of avian and mammalian influenza viruses [32]. However, the precise roles of individual importin α isoforms in NP nuclear transport and replication of influenza viruses remain to be clarified.

This study examined the contribution of importin α isofoms for NP localization to the nucleus and viral multiplication via the unconventional NLS and found that Qip1 is responsible for S9A virus multiplication independent of nuclear localization. This conclusion was reached through the following steps: (i) We evaluated the effects of mutating the unconventional NLS between residues 3 and 13 on the nuclear localization of NP. (ii) To analyze the relationship between nuclear localization and binding with importin α isoforms, we performed a glutathione-S-transferase (GST)-pull down assay and immunoprecipitation assay using unconventional NLS mutants, which showed different characteristics in terms of nuclear localization. Given the possible effect of the two bipartite NLSs on the affinity for importin α, we used only the N-terminal 110-amino acid region (NP110aa) lacking half of the overlapping bipartite NLS (spanning amino acids 90 through 121) and the entire bipartite NLS (spanning amino acids 198 through 216); thus, the mutant NP lacked all nuclear localization function of two bipartite NLSs. (iii) We identified the contribution made by two amino acids, Arg 8 and Ser 9, within the unconventional NLS (which show different conservation rate in human and avian influenza A viruses) on viral replication, vRNA transcription. (iv) Finally, we knocked-down Qip1 in HEK-293 and A549 cells to identify whether Qip1 is required for viral replication and vRNA transcription.

## Results

### The amino acid at position 8 is the major site determining the nuclear localization of NP in A549 cells

**Figure 1:**
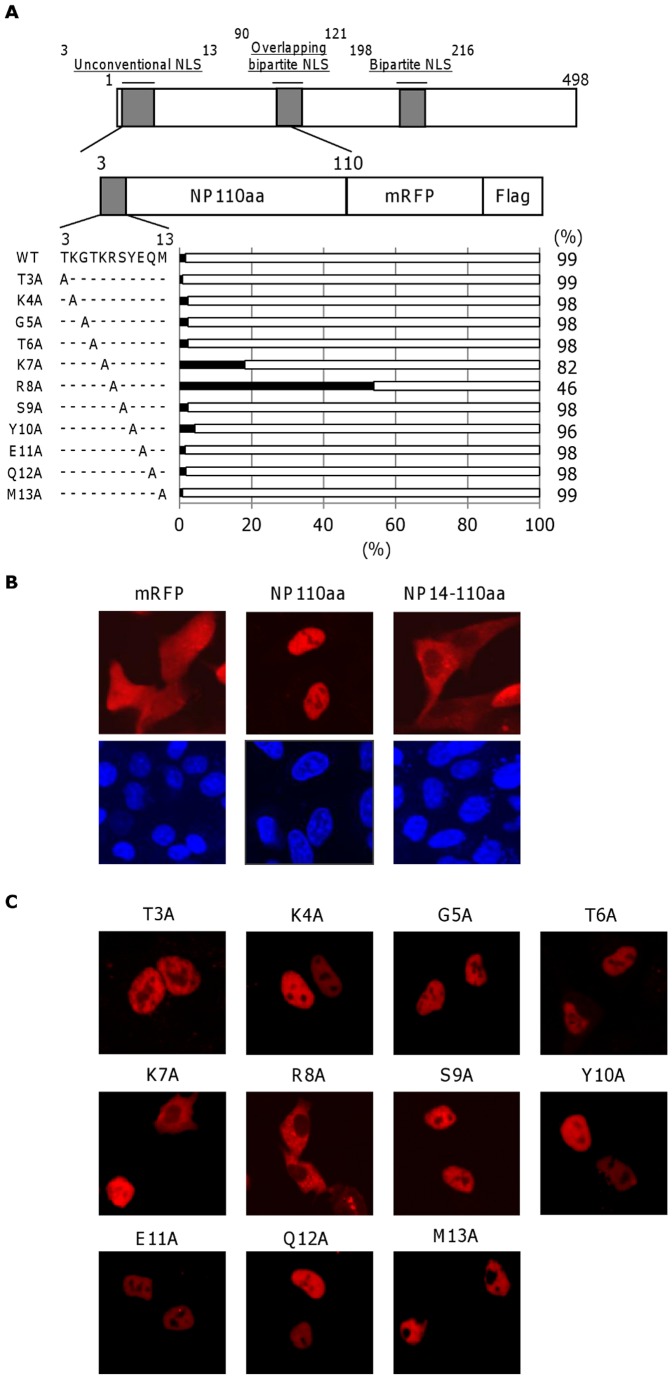
Subcellular localization of unconventional NLS mutants. (A) Schematic diagram and subcellular localization of the N-terminal 110-amino acid region (NP110aa) NLS mutants. The unconventional NLS (aa 3-TKGTKRSYEQM-13), overlapping bipartite NLS (aa 90-KKTGGPIYRRVDGKWRRELILYDKEEIRRIWR-121) or bipartite NLS (aa 198-KRGINDRNFWRGENGRKTR-216) are indicated in gray in the schematic showing of full length NP and NP110aa. A549 cells were transfected with plasmids expressing WT and mutant NP110aas. The nuclear and cytoplasmic localization of WT and mutant NP110aas were determined by monitoring mRFP fluorescence. Over five hundred mRFP-expressing cells were counted and classified as nuclear or cytoplasmic localization. (B) Fluorescence microscope images showing mRFP, WT NP110aa and a mutant lacking the N-terminal 13-amino acid tail region (NP14-110aa) (upper panel). Nucleus is stained with DAPI (lower panel). (C) Fluorescence microscope images showing WT and mutant NP110aas. The localization of NP was consistent with that observed using mRFP fluorescence.

To examine whether the unconventional NLS between residues 3 and 13 is involved in the nuclear localization function of NP, we constructed N-terminal 110-amino acid region of NP (NP110aa) and a mutant lacking the N-terminal 13-amino acid tail region (NP14-110aa) plasmids with the mRFP and a Flag tag at the carboxyl-terminus (Fig. 1A) [33]. NP110aa lacks half of the overlapping bipartite NLS spanning amino acids 90 through 121 and the entire bipartite NLS spanning amino acids 198 through 216. The expression vectors encoding NP110aa and NP14-110aa were transfected into A549 cells and the expression of each protein was examined by confocal laser-scanning microscopy (Fig. 1B). NP110aa localized primarily within the nucleus (visible as speckles), and NP14-110aa mainly localized to the cytoplasm, indicating that the unconventional NLS had nuclear localization activity.

We next identified the amino acid position necessary for nuclear localization by mutating the alanine residues within the unconventional NLS in NP110aa (Fig. 1A and C). The subcellular localization of the substitution mutants was determined by monitoring mRFP fluorescence in the nucleus and cytoplasm (Fig. 1A and C). Nine of the mutants (the exceptions were R8A NP110aa and K7A NP110aa) mainly localized in the nucleus 48 h after transfection into A549 cells. Expression of K7A NP110aa was observed in the nuclei of 82% of transfected cells, and showed cytoplasmic staining in only 18% (Fig. 1A). By contrast, the R8A NP110aa mutant showed only diffuse cytoplasmic staining in 54% of cells. These data clearly show that amino acid residue 8 within the unconventional NLS is associated with the nuclear localization of NP.

### Viral production is reduced after the mutation of amino acid position 8 within the unconventional NLS

**Figure 2:**
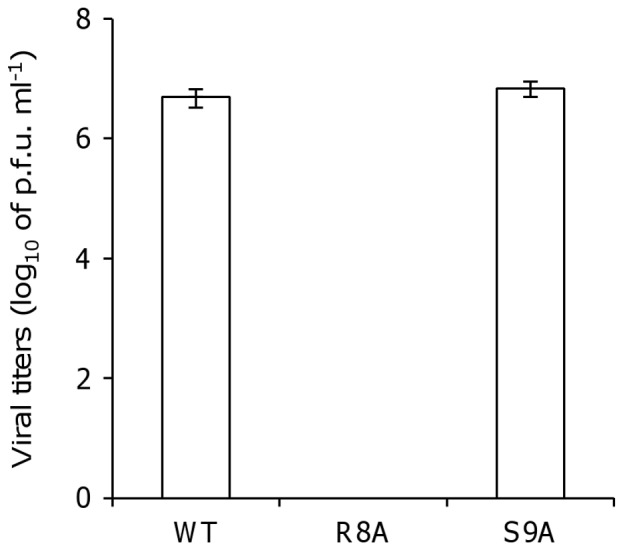
Production of recombinant viruses expressing unconventional NLS mutants. HEK-293T/MDCK cells were transfected with plasmids expressing four viral proteins and eight vRNAs. To generate the R8A NP and S9A NP mutant viruses, plasmids expressing mutant NP proteins and genomes were used as a substitute for the WT plasmid. After 72 h of transfection, supernatants were harvested and subjected to plaque assays using MDCK cells. Values represent the mean ± SD of measurements taken from three samples in each of four independent experiments.

To demonstrate the role of the unconventional NLS of NP on viral production, we generated mutant viruses with amino acid substitutions R8A or S9A using a reverse-genetics approach (Fig. 2). We harvested only wild-type virus when we performed the reverse-genetics using a plasmid encoding mutant NP vRNA and a support plasmid encoding wild-type NP. Therefore, in subsequent experiments, we performed reverse-genetics with support plasmids encoding mutant NPs, whose nucleotide sequences were identical to the open reading frames of the plasmids encoding the mutant NP vRNAs.

As a result, the recombinant virus possessing the R8A NP mutant, which showed a 50% reduction in NP nuclear localization activity, was still not rescued at 72 h after transfection into HEK-293T/MDCK cells. However, virus possessing the S9A NP mutation, which predominantly localized in the nucleus, was able to generate progeny at the same level as the WT NP virus (5×10^6^ PFU/ml *vs.* 7×10^6^ PFU/ml, respectively) (Fig. 2). This result clearly shows that the production of mutant virus was completely inhibited by the R8A mutation, which also reduced NP nuclear localization activity.

### Amino acid residue 9 is involved in viral replication and vRNA transcription

**Figure 3:**
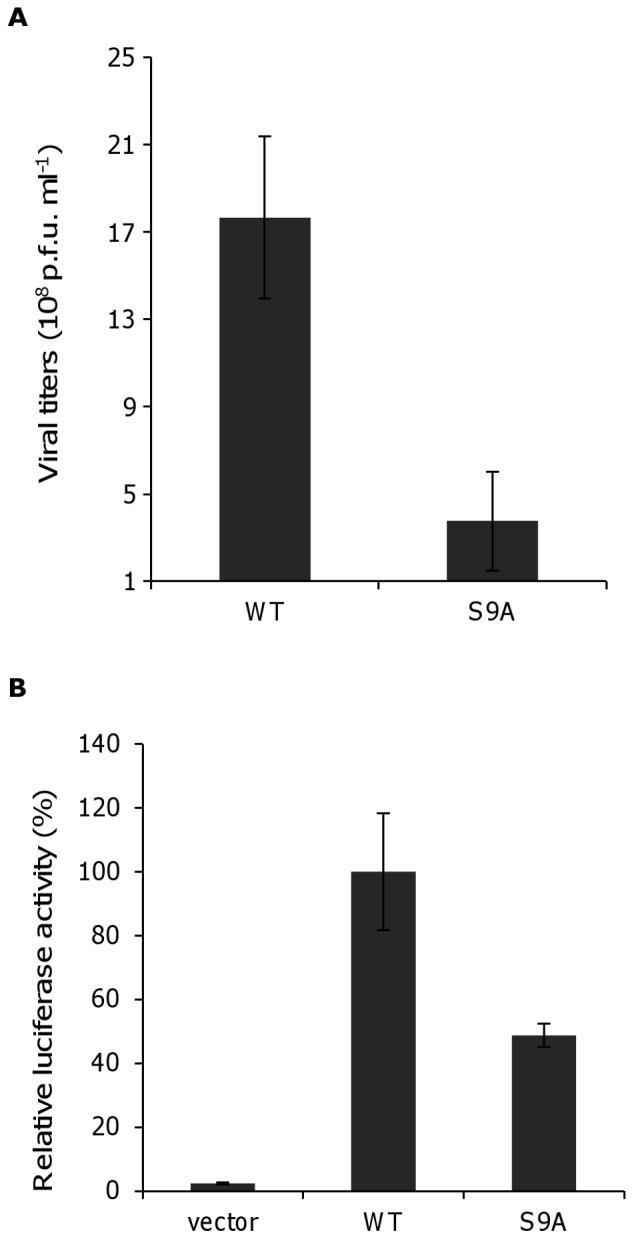
Effect of unconventional NLS mutants on viral replication and vRNA transcription. (A) A549 cells were infected with the S9A NP mutant and WT NP viruses at a multiplicity of infection of 1. At 24 h post-infection, the supernatants were harvested and the virus titer was determined using plaque assays. Data represent the relative growth of the S9A mutant compared with that of the WT. (B) Mini-genome assay using the S9A NP mutant. The effect of the S9A mutation on viral transcription was determined using a mini-genome assay with the plasmid encoding the S9A NP mutant. Luciferase activity was measured 48 h after the transfection of viral protein- (PB2, PB1, PA or NP) and vNP-luc-expressing plasmids. To analyze the effect of the S9A mutation on vRNA transcription, luciferase activity was compared with that generated by the WT. Data represent the mean ± SD of measurements from three samples in each of two independent experiments (*p<0.05, by students *t*-test).

To examine the role of amino acid residue 9 on the replication of influenza virus, A549 cells were infected with S9A NP mutant or WT NP viruses (produced by reverse-genetics) at a multiplicity of infection of 1 (MOI 1), and the viral titers were determined using a plaque assay 24 h later. The growth of S9A NP mutant virus was attenuated compared with that of the WT virus (4×10^8^ PFU/ml *vs.* 1.8×10^9^ PFU/ml, respectively) (Fig. 3A). Because S9A NP110aa mainly localized to the nucleus, the reduction in viral growth observed for the S9A mutant may be due to other functions of the unconventional NLS, which do not relate to nuclear transport.

To confirm this hypothesis, we analyzed the effect of mutating amino acid residue 9 on the transcription of vRNA using a mini-genome assay (Fig. 3B). Plasmids encoding WT NP or S9A NP were transfected into HEK-293 cells together with expression plasmids encoding the viral polymerase subunits and a vNP-luc/pHH21 plasmid. Despite the finding that S9A NP localized to the nucleus at the same level as WT-NP, the luciferase activity generated by S9A NP was only half that generated by WT NP after 48 h. These results clearly indicate that the unconventional NLS of NP is involved in the transcription of influenza virus vRNA in addition to its nuclear transport activity, although both functions appear to operate via independent pathways.

### Amino acid residue 8 is fully conserved in human and avian influenza A viruses

To investigate the conservation of amino acid residues 8 and 9 between influenza A viruses of human and avian origin, NP sequences obtained from the NCBI Influenza Virus Resource (http://www.ncbi.nlm.nih.gov/genomes/FLU/FLU.html) were analyzed using perl script (Table 1). No mutation of amino acid residue 8 was identified in 6,748 human and 3,017 avian influenza NP sequences, indicating that the Arg residues at position 8 may be essential for influenza virus multiplication. However, mutations were observed in the Ser residue at position 9 in three human and 13 avian sequences; the proportion of conserved amino acids was about 99% for both species, indicating that Ser residues at position 9 may influence the viability of influenza virus.

**Table 1:** Conservation of 8 and 9 amino acid residue in human and avian influenza A virus.

		Mutation	
Position	Amino acid	Human	Avian
8	R	0	0
9	S	3	13

Total 6,748 sequences of human NP were analyzed by perl script. Total 3,017 sequences of avian NP were analyzed by perl script.

### The preferential binding of unconventional NLS within NP to Qip1

**Figure 4:**
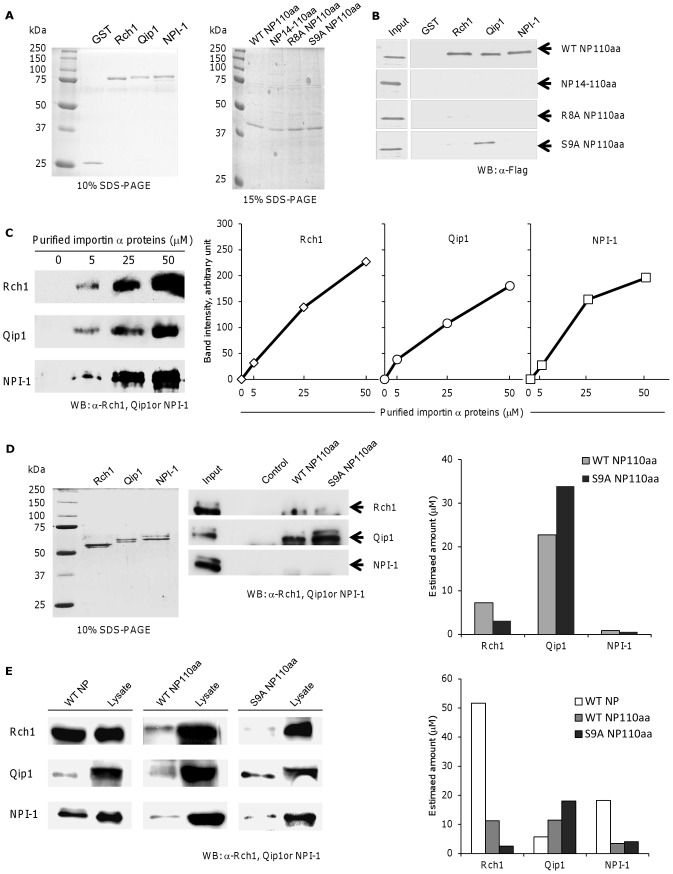
Binding of NP to importin α. (A) Purification of mRFP-Flag-tagged WT and mutant NP110aas from COS-7 cells and GST-tagged importin α isoforms, Rch1, Qip1 and NPI-1 from *Escherichia coli*. For purification of NP110aa, COS-7 cells were transfected with pCAGGS encoding mRFP-Flag-tagged WT and mutant NP110aas, lysed and proteins were purified using ANTI-FLAG M2 Agarose beads and the 3× Flag peptide. For purification of importin α proteins, Escherichia coli expressing GST-tagged Rch1, Qip1 and NPI-1 were lysed and proteins were purified using glutathione-Sepharose 4B. Purified proteins were subjected to 10% or 15% SDS-PAGE and stained by Coomassie brilliant blue. (B) Binding of WT and mutant NP110aas to the three importin α isoforms. Both GST beads bound to Rch1, Qip1, NPI-1 and non-bound GST beads were incubated with the WT, 14-110aa, R8A or S9A NP110aa proteins. Binding of NP110aa to importin α was detected by Western blotting with anti-FLAG M2 monoclonal antibody (mAb). (C) Quantification of band intensity. Different concentrations of Rch1, Qip1 and NPI-1 proteins (0, 5, 25 and 50 µM) were subjected to 10% SDS-PAGE and Western blotting with anti-Rch1, -Qip1 and -NPI-1 mAbs. Band intensities were measured by Image J and the standard curves for the amount of importin α isoforms were constructed. (D) Competition binding assay of importin α to WT and S9A NP110aa-binding beads. Purified importin α isoforms, Rch1, Qip1 and NPI-1 from *Escherichia coli* were subjected to 10% SDS-PAGE and stained with Coomassie brilliant blue. All three importin α proteins (50 µM) were incubated with WT and S9A NP110aa-binding Flag beads at 4°C for 1 day. After washing with buffer, the binding of importin α to WT and S9A NP110aa was detected by Western blotting with anti-Rch1, -Qip1 and -NPI-1 mAbs. Empty Flag beads were used as the negative control. The amount of importin α proteins which bound to WT NP110aa and S9A NP110aa were calculated by analyzing the standard curves as shown in C right panel. (E) Binding of WT full length NP, WT and S9A NP110aa to the three importin α isoforms in HEK-293T cells. HEK-293T cells were transfected with pCAGGS encoding mRFP-Flag-tagged WT full length NP, WT or S9A NP110aa, cells were lysed and proteins were purified using ANTI-FLAG M2 Agarose beads and the 3× Flag peptide. The bound importin αs to NP was detected by Western blotting with anti-Rch1, -Qip1 and -NPI-1 mAbs. The same amount of lysate was used for control. The amounts of importin α proteins which bound to WT NP, WT NP110aa and S9A NP110aa were calculated by analyzing the standard curves as shown in C right panel.

Although NP binds to Rch1, Qip1 and NPI-1 [24], the regions of NP responsible for the binding activity are unknown. To determine whether importin α is involved in the function of the unconventional NLS of NP, we determined its binding specificity for each of the three importin α isoforms. Recombinant GST-tagged importin α isoforms (Rch1, Qip1 and NPI-1) were immobilized on glutathione-Sepharose beads and incubated with mRFP-Flag-tagged NP110aa or its mutants NP14-110aa, R8A NP110aa and S9A NP110aa followed by purification from vertebrate cells (Fig. 4A and B). WT NP110aa was able to interact with all three importin α isoforms almost equally. Interestingly, this binding was markedly reduced by deletion of the unconventional NLS between residues 3 to 14 (NP 14-110aa), indicating that all of the three importin α binding sites are located in the unconventional NLS. We next analyzed the effect of amino acid substitutions within the unconventional NLS on binding to the three importin α isoforms. R8A NP110aa lost the ability to bind Qip1 and NPI-1 but still showed very weak binding to Rch1. By contrast, the interaction between S9A NP110aa and Qip1 was almost the same as that observed for WT NP110aa; however, it bound very weakly to Rch1 and NPI-1 (Fig. 4B).

The present study indicates that the unconventional NLS is the main region of NP that binds the three importin α isoforms. Therefore, we next examined the binding affinity of WT NP110aa and S9A NP110aa for the three importin α isoforms using a compatitive binding assay. To quantify the importin α proteins which bound to NP, we measured band intensities which were observed after Western blot analysis (Fig. 4C, left panel) and made the standard curve for the amount of importin α isoforms (Fig. 4C, right panel). When all three importin α proteins were incubated with WT or S9A NP110aa-conjugated beads, subsequent Western blot analysis detected bindings of WT and S9A NP110aa to Qip1 (Fig. 4D, left panel). These binding amounts were measured by analyzing the standard curves as shown in Fig. 4C, right (Fig. 4D, left). By contrast, these bindings were lost on NPI-1, but faint bindings were still observed on Rch1.

As shown in Fig. 4E, right and left panels, the binding of Rch1 and NPI-1 to WT NP110aa were notably decreased comparing with those of WT full-length NP in live cells. In addition, S9A mutation in unconventional NLS of NP110aa resulted in obvious reduction in affinity bindings for Rch1. By contrast, the bindings of WT and S9A NP110aa to Qip1 did not reduced comparing with WT full length NP.

Taken together, Qip1 preferentially bound to S9A NP110aa comparing with Rch1 and NPI-1 in *vitro* and *vivo*.

### Qip1 is important for transcription and growth of S9A virus via a nuclear localization-independent mechanism

**Figure 5:**
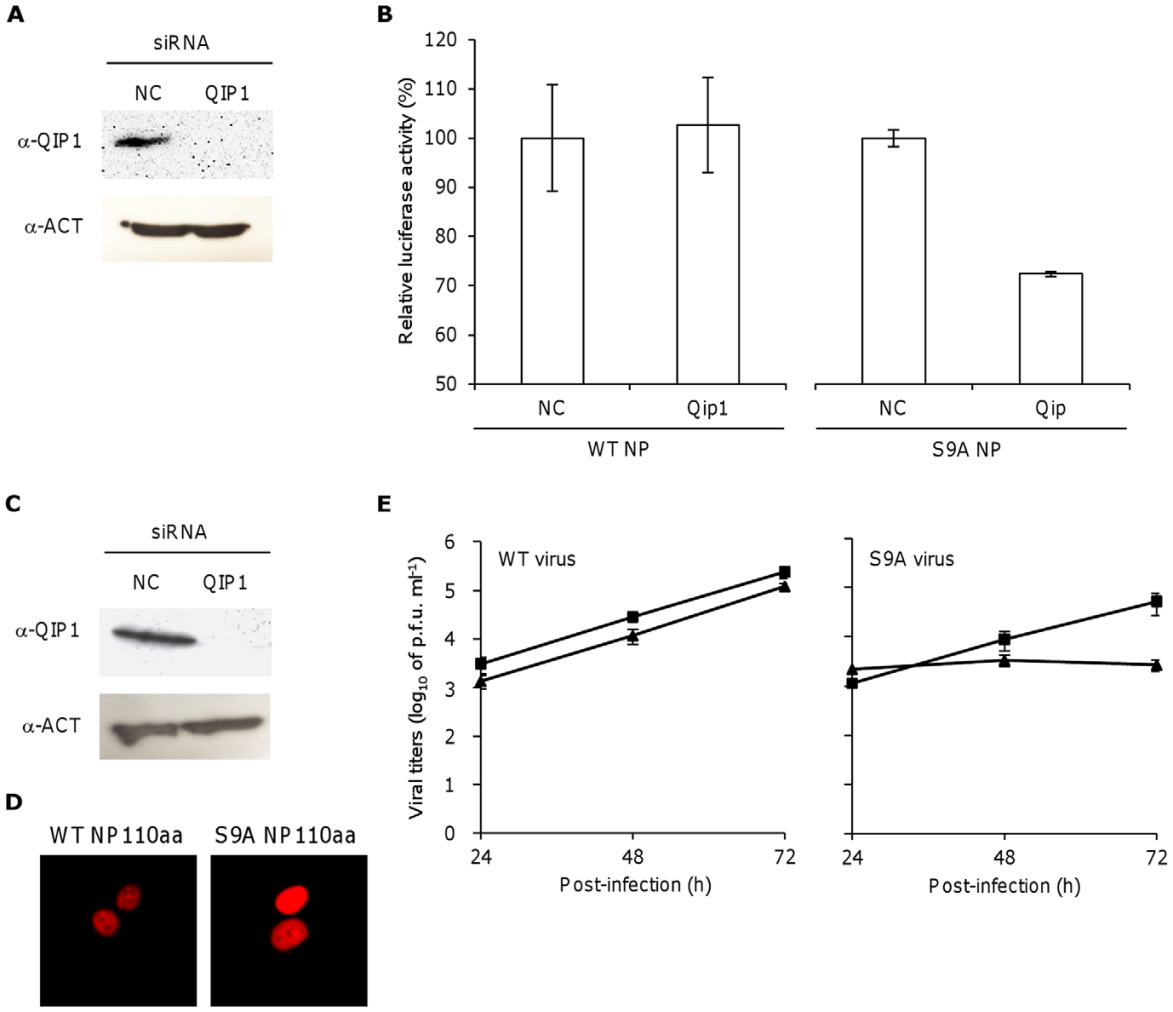
Viral transcription and growth in Qip1-silenced cells. (A) HEK-293 cells were transfected with Qip1 siRNA for 24 h. Silencing of Qip1 was confirmed by Western blotting with an antibody specific for Qip1. (B) Qip1-silenced HEK-293 cells were transfected with plasmids encoding PB2, PB1, PA, WT or S9A NP and vNP-luc, and luciferase activity was measured after 48 h. Columns and error bars represent the mean ± SD of measurements from three samples in each of three independent experiments (*p<0.05, by students *t*-test). (C) A549 cells were transfected with Qip1 siRNA for 48 h. Silencing of Qip1 was confirmed by Western blotting with an antibody specific against Qip1. (D) Qip1-silenced A549 cells were transfected with pCAGGS encoding mRFP-Flag-tagged WT or S9A NP110aa. The localization of NP was consistent with that observed using mRFP fluorescence. (E) The Qip1-silenced A549 cells were infected with A/WSN/33 virus containing WT or S9A NP at an MOI of 0.01. The viral titer in supernatant of non-targeting siRNA transfected (triangle) or Qip1 siRNA transfected (square) cells was determined using a plaque assay. Data represent the mean ± SD of measurements from three samples in each of three independent experiments (*p<0.05, by students *t*-test).

To determine whether Qip1 is involved in viral multiplication, viral transcription and replication of the WT and the S9A NP mutant were examined in A549 cells and in HEK-293 cells in which Qip1 expression had been knocked down (Fig. 5). Transfection of HEK-293 cells with siRNA targeting Qip1 fully silenced Qip1 expression after 24 h with no associated changes in the level of beta-actin expression (Fig. 5A). The effect of Qip1 silencing on the transcription of vRNA was then examined in a mini-genome assay. There were no significant differences in luciferase activities between cells treated with Qip1 siRNA and those negative control siRNA using WT NP plasmid. The luciferase activity was reduced by approximately 50% in negative control siRNA treated cells using S9A NP plasmid compare to WT NP plasmid. Furthermore, luciferase activity was significantly reduced by approximately 30% in Qip1-silenced cells compared to negative control siRNA treated cells using S9A NP plasmid (p<0.05) (Fig. 5B).

Next, we determined the nuclear localization of WT and S9A NP110aa in A549 cells, in which Qip1 had been knocked-down. Silencing of Qip1 was confirmed by Western blot analysis 2 days after transfection with Qip1-specific siRNA but not after transfection with negative control siRNA, without observing associated changes in beta-actin expression (Fig. 5C). The nuclear localization of WT and S9A NP110aa was not changed by Qip1 silencing (Fig. 5D). Furthermore, we examined the viral growth of the WT and the S9A mutant virus in Qip1-silenced cells. The amounts of WT and S9A mutant viruses in the cell culture supernatant were measured after 24, 48 and 72 h of infection using a plaque assay (Fig. 5E). Although there were no notable differences in the growth of the WT virus between Qip1-silenced cells and control cells, the titer of WT virus was consistently lower in Qip1-silenced cells at 24, 48 and 72 h post infection. In the S9A virus, the viral titer was similar to those of control and Qip1-silenced cells (1.1×10^3^ PFU/ml *vs.* 2.2×10^3^ PFU/ml, respectively) after 24 h. At 48 h post-infection, the viral titer was slightly higher in control cells than in Qip1-silenced cells (8.9×10^3^ PFU/ml *vs.* 3.4×10^3^ PFU/ml, respectively). Interestingly, viral growth was significantly impaired in Qip1-silenced cells at 72 h infection (p<0.05) (5.2×10^4^ PFU/ml *vs.* 2.7×10^3^ PFU/ml for control and Qip1-silenced cells, respectively).

## Discussion

The results of the present study lead to four major conclusions. First, the amino acid at position 8 within the unconventional NLS of NP is necessary for nuclear localization and viral multiplication. This was confirmed by showing that the conservation of amino acid residue 8 of NP is necessary for the propagation of both human and avian influenza viruses. Second, the mutant S9A NP110aa, which mainly localized to the nucleus, showed reduced viral growth and vRNA transcription. This indicated that the unconventional NLS of NP is likely associated with vRNA transcription and viral growth, albeit via independent pathways. Third, NP110aa, which was localized to the nucleus, interacted with all three importin α isoforms (Rch1, NPI-1, and Qip1) at a similar level. Indeed, the binding and nuclear localization were markedly reduced after deletion of the unconventional NLS. This is supported by data showing that R8A NP110aa, which lost nearly 50% of its nuclear localization activity, had decreased binding for Qip1, NPI-1 and Rch1. Lastly, we showed that, although binding of Rch1 and NPI-1 was reduced in WT NP110aa and S9A NP110aa compare to full-length WT NP, Qip1 bound to WT NP110aa and S9A NP110aa as well as full-length WT NP. However, viral replication and vRNA transcription were reduced by Qip1 silencing only in S9A NP. Furthermore, nuclear localization of WT and S9A NP110aa was unaffected by Qip1 silencing. Thus, Qip1 appears to contribute to S9A NP virus multiplication through the unconventional NLS independently of nuclear localization of NP, whereas it is not essential for multiplication of WT virus because it may hide under interaction of many importin isoforms to WT NP.

This study also shows that, although S9A NP110aa mainly localized to the nucleus, viral growth and vRNA transcription were reduced by the S9A mutation, indicating that the amino acid at position 9 within the unconventional NLS may have an important function during viral multiplication aside from nuclear localization. However, the reduction in viral titers of S9A mutant was not detected in Fig. 2. The reason of this discrepancy may be due to the duration of experimental period of virus production. In Fig. 2, we determined whether R8A and S9A mutant viruses are able to rescue by reverse-genetics. In this case of virus-plasmids-transfected cells, many cells died by viral cytopathic effect in both WT and S9A viruses when we cultured over 72 hours. Thus, the generation of progeny viruses may saturate at 72 hours post transfection, thereby resulting in equivalent titer between WT and S9A mutant viruses. To examine the effect of the mutation at S9A to viral growth in Fig. 3, we performed a preliminary examination and then obtained results that virus-infected cells at moi of 1 were died at 72 hours infection, indicating that 72 hours in infection time of moi of 1 may be a long time and should cut short this lengthy incubation time. Indeed, we obtained differences the titer of mutant virus with S9A when we evaluated the production of progeny virus at 24 hours post infection. Thus, the attenuation of viral growth may become clear in Fig. 3. These results indicate that the unconventional NLS of NP is involved in viral multiplication and nuclear transport via two distinct pathways. Interestingly, it appears that in PB2, the NLS is associated with vRNA replication in a nuclear localization-independent manner [34]. Furthermore, our mutational analysis studies suggest that nine mutants containing alanine mutations within the unconventional NLS retained the ability to translocate to the nucleus and, in addition, all nine amino acids are conserved in approximately 99% of human- and avian-origin influenza viruses (data not shown). These results suggest that other amino acids (in addition to the amino acid at position 9) within the unconventional NLS may be associated with viral multiplication independent of nuclear localization activity.

Previously, it was reported that the NP binds to Rch1, NPI-1 and Qip1 [18], [24], and interactions between NP, Rch1 and NPI-1 observed in a yeast two-hybrid assay correlate well with the nuclear localization of NP [24]. The present study shows that NP14-110aa, which has lost nuclear localization capacity, also lost the ability to bind all three importin α isoforms. This clearly indicates that the binding of importin α by the unconventional NLS is essential for nuclear localization. In addition, binding of all three importin α isoforms to R8A NP110aa was markedly reduced in a similar manner. Considering that both mutants mainly localized to the cytoplasm, binding of importin α to amino acid residue 8 appears to be required to initiate the nuclear localization function of the unconventional NLS of NP. Interestingly, we also found that S9A NP110aa had a preference binding to Qip1. Binding of Rch1 or NPI-1 to S9A NP110aa was markedly reduced compared with that shown by WT NP110aa. In contrast to Rch1 and NPI-1, binding of Qip1 to S9A NP110aa was almost the same as that observed for WT NP110aa (Fig. 4B). Furthermore, Qip1 out-competed Rch1 and NPI-1 for binding to WT NP110aa and S9A NP110aa (Fig. 4D). These results indicate that unconventional NLS of NP mainly bound to Qip1. A previous report shows that the mutation of the ARM repeat 8 within NPI-1 and Qip1 reduced binding to NP, whereas binding was still detectable after the same mutation was introduced into Qip1 [18]. This indicates that the binding site of Qip1 may span a larger number of amino acids compared to NPI-1. These differences may contribute to the binding of Qip1 to S9A NP110aa. Furthermore, we determined the binding of NP to Rch1, Qip1 and NPI-1 in live cells by immunoprecipitation assay (Fig. 4E). Using the signal in lysate detected by western blot as the baseline, the binding of WT NP110aa and S9A NP110aa to Rch1 and NPI-1 was reduced compare to full-length WT NP. On the other hand, Qip1 bound to WT NP110aa and S9A NP110aa as well as full length WT NP. These results may indicate that Qip1 is preferentially bound to unconventional NLS of NP.

Influenza viruses derived from different hosts utilize different importin α isoforms. The avian influenza virus (A/FPV/Rostock/1/34 (H7N7)) uses Qip1 for efficient virus replication, whereas human influenza virus (A/Victoria/3/75 (H3N2)) uses importin α7 [32]. As an illustration of the human adaptation of avian viruses in swine, the 2009 pandemic viruses (A/Sachsen-Anhalt/101/09 (H1N1) and A/Hamburg/NY1580/09 (H1N1)) utilized both Qip1 and importin α7 for efficient viral replication [32]. We also examined the importance of Qip1 binding to NP for viral transcription and growth using WT or S9A NP mutant and Qip1-silenced A549 cells. vRNA transcription by WT NP WSN (H1N1) in Qip1-silenced cells did not show difference that in cells which treated with negative control siRNA. However, vRNA transcription by S9A NP WSN in Qip1-silenced cells decreased to around 70% of that in control cells (Fig. 5A and B). The kinetics of WT virus growth kept a tendency to reduce in Qip1-silenced cells as compared with control cells during 72 h post-infection, whereas notable difference of viral growth also was not determined between control and Qip1-silenced cells. On the other hand, viral growth of the S9A mutant was notably reduced at 72 h post-infection in Qip1-silenced A549 cells compared to that in control cells (Fig. 5C and E). This indicates that the replication of S9A mutant virus requires Qip1. Because viral growth of the WT virus through importin αs can be complemented by many isoforms, replication and viral growth of the WT virus was not affected by Qip1 knock down. Indeed, transcription and viral growth were reduced using S9A mutant NP, which was attenuated the binding activity of NP to Rch1 and NPI-1. This may suggest that other factors, such as Rch1, NPI-1 or importin α7 contribute to multiplication of the virus. Therefore, further studies are required to understand in detail the mechanism of how Qip1 is involved in the replication of human-origin H1N1 virus via cooperation with other importin α family members.

Many studies show that the unconventional NLS is indispensable for nuclear localization of NP [11], [12], [24], [30]. In particular, amino acid residues 7 and 8 are critical for the nuclear localization of NP in COS-7 and HeLa cells [24], [30]. In the present study, we also confirmed that the amino acids at positions 7 and 8 are associated with nuclear localization of NP in A549 cells, which are susceptible cells to infection by influenza A virus. However, the percentage of cells showing cytoplasmic localization was clearly higher in R8A NP110aa-transfected cells than in K7A NP110aa-transfected cells. This clearly indicates that amino acid residue 8 is the major site for nuclear localization within NP. However, although nuclear localization of R8A NP110aa was still almost 50% of analyzed cells, viruses with the R8A mutation were not rescued by reverse-genetics. Ozawa *et al.* (2007) indicated that the unconventional NLS of NP is associated with both the nuclear transport of NP and packaging of vRNA [31]. Furthermore, disruption of nuclear localization by mutating the unconventional NLS of NP led to a reduction in vRNA transcription [31]. Therefore, suppression of nuclear localization, vRNA transcription, and packaging of vRNA by the R8A mutation may result in the inability to rescue R8A mutant viruses. Interestingly, amino acid residue 8 of NP is fully conserved in both human- and avian-origin influenza viruses. These results indicate that the amino acid residue 8 of NP is essential for viral multiplication.

In conclusion, the results of the present study show for the first time that importin α3/Qip1 contribute to the proper function of the unconventional NLS of S9A NP, including up-regulation of transcription and/or replication of vRNA, and viral multiplication in a nuclear localization independent manner. Furthermore, we showed that, although S9A NP110aa mainly localized to the nucleus, viral growth and vRNA transcription was reduced by the S9A mutation, indicating that the unconventional NLS of NP is involved in vRNA transcription and nuclear transport via two distinct pathways.

## Materials and Methods

### Cells and viruses

Madin-Darby canine kidney (MDCK) cells [33], human lung carcinoma A549 cells (ATCC, cat no: CCL-185), African green monkey kidney COS-7 cells [33], human embryonic kidney HEK-293 cells (ATCC, cat no: CRL-1573) and HEK-293T cells [35] were cultured in Dulbecco's modified Eagle's medium (Sigma) containing Pen Strep Glutamine (PSG, Gibco) and 10% fetal bovine serum (Sigma). All cells were maintained at 37°C in 5% CO_2_. The influenza A/Wilson-Smith/1933 (A/WSN/33, H1N1) virus strain [36] was propagated in MDCK cells at 37°C for 2 days in 5% CO_2_.

### Construction of plasmids

The GST expression vector pGEX-6P-3, encoding GST-tagged-Rch1, -Qip1 or -NPI-1 (19), and the mammalian expression vector pCAGGS [37], encoding the monomeric red fluorescent protein (mRFP)-Flag-conjugated N-terminal 110-amino acid region of NP (NP110aa) or a deletion of the unconventional NLS located within the N-terminal 13-amino acids of NP110aa (NP14-110aa) [33], have been described previously. pCAGGS encoding mRFP-Flag conjugated alanine mutants of the unconventional NLS extending from amino acids 3 to 13 of NP (T3A-, K4A-, G5A-, T6A-, K7A-, R8A-, S9A-, Y10A-, E11A- Q12A and M13A-NP110aa), were generated by PCR using the following forward primers: *XhoI*-T3A NP fwd, 5′-AAACTCGAGATGGCGGCCAAAGGCACCAAACGA-3′; *XhoI*-K4A NP fwd, 5′-AAACTCGAGATGGCGACCGCAGGCACCAAACGA-3′; *XhoI*-G5A NP fwd, 5′-AAACTCGAGATGGCGACCAAAGCCACCAAACGA-3′; *XhoI*-T6A NP fwd, 5′-AAACTCGAGATGGCGACCAAAGGCGCCAAACGATCT-3′; *XhoI*-K7A NP fwd, 5′-AAACTCGAGATGGCGACCAAAGGCACCGCACGATCTTAC-3′; *XhoI*-R8A NP fwd, 5′-AAACTCGAGATGGCGACCAAAGGCACCAAAGCATCTTACGAA-3′; *XhoI*-S9A NP fwd, 5′-AAACTCGAGATGGCGACCAAAGGCACCAAACGAGCTTACGAACAG-3′; *XhoI*-Y10A NP fwd, 5′-AAACTCGAGATGGCGACCAAAGGCACCAAACGATCTGCCGAACAGATG-3; *XhoI*-E11A NP fwd, 5′-AAACTCGAGATGGCGACCAAAGGCACCAAACGATCTTACGCACAGATGGAG-3′ and *XhoI*-Q12A NP fwd, 5′-AAACTCGAGATGGCGACCAAAGGCACCAAACGATCTTACGAAGCGATGGAGACT-3′; *XhoI*-M13A NP fwd, 5′-AAACTCGAGATGGCGACCAAAGGCACCAAACGATCTTACGAACAGGCGGAGACT-3′; and the reverse primer: *Spe*-NP110aa-R, 5′-AAAACTAGTAAGGATGAGTTCTCTCCTCC-3′ (restriction enzyme sites underlined). The viral genome-expressing plasmids PB2/pHH21, PB1/pHH21, PA/pHH21, HA/pHH21, NP/pHH21, NA/pHH21, M/pHH21, NS/pHH21 and empty-pHH21 [38], [39]; the expression plasmids PB1/pCAGGS, PB2/pCAGGS, PA/pCAGGS and NP/pCAGGS; and the vNP-luc/pHH21 plasmid derived from the A/WSN/33 (H1N1) virus were kind gifts from Dr. Y Kawaoka, University of Tokyo. To generate the plasmids expressing alanine-mutated viral NP genomes (R8A vNP/pHH21 and S9A vNP/pHH21), PCR was performed using NP/pHH21 as the template with KOD Plus Ver. 2 (TOYOBO) and the following primers: *BsmBI*-R8A vNP fwd, 5′-CGTCTCNGGGAGCAAAAGCAGGTCACTCACAGAGTGACATCGAAATCATGGCGACCAAAGGCACCAAAGCATCTTACGAACAGATG-3′; *BsmBI*-S9A vNP fwd, 5′-CGTCTCNGGGAGCAAAAGCAGGTCACTCACAGAGTGACATCGAAATCATGGCGACCAAAGGCACCAAACGAGCTTACGAACAGATG-3′; and *BsmBI*-vNP rev, and 5′-CGTCTCNTATTAGTAGAAACAAGGTTCTTTAA-3′. After purification, the PCR products were digested with *BsmBI* (New England Biolabs) and cloned into the *BsmBI* sites within the pHH21 vector [38]. To produce pCAGGS encoding the full-length NP mutants (R8A-NP/pCAGGS and S9A-NP/pCAGGS), PCR amplification was performed using KOD Plus Ver. 2 (Toyobo) with a full-length first-strand NP cDNA as the template and the forward primers *XhoI*-R8A NP fwd or *XhoI*-S9A NP fwd, and the reverse primer rev: 5′-AAAGCGGCCGCTTAATTGTCGTACTCCTCT-3′. The PCR products were cloned into the *Xho*I and *Not*I sites within pCAGGS.

### Expression and confocal laser-scanning microscopic analysis of the mRFP-Flag fusion proteins

A549 cells (7×10^5^ cells) were transfected with 2 µg of pCAGGS encoding mRFP-Flag-conjugated NP110aa, NP14-110aa, T3A-, K4A-, G5A-, T6A-, K7A-, R8A-, S9A-, Y10A-, E11A-, Q12A- or M13A-NP110aa using the FuGENE HD Transfection Reagent (Roche). Two days after transfection, the expressed proteins were observed under a confocal laser-scanning microscope (FV 1000, Olympus).

### Plaque assay

A549 cells were infected with A/WSN/33 (H1N1) virus or the recombinant viruses. After adsorption at 37°C for 1 h, cells were washed three times with phosphate-buffered saline (PBS) and incubated in serum-free medium at 37°C. The supernatant was collected at the specified time points and subjected to a plaque assay. For the plaque assay, MDCK cells (6×10^5^) were cultured in 6 well plates at 37°C for 1 day. After washing with PBS, cells were infected with influenza virus for 1 h. After washing again, cells were incubated in 2 ml of modified Eagle's medium (MEM) (2×) containing 1% agarose with 1 µg/ml of tosylsulfonyl phenylalanyl chloromethyl ketone-treated trypsin (Trypsin-TPCK, Worthington Biochemical Corporation). The cells were then incubated at 37°C for 2 days in 5% CO_2_. The number of plaque spots was counted and the virus titer was calculated.

### Generation of recombinant viruses

Recombinant viruses were generated by DNA transfection as described previously [39], [40]. Briefly, a co-culture of MDCK (6×10^5^) and HEK-293T (3×10^5^) cells was transfected with eight viral genome-expressing plasmids or four viral protein-expressing plasmids using the FuGENE HD transfection reagent (Roche). At 72 hours post-transfection, the recombinant viruses were harvested and the viral titer in the cell supernatants was determined using a plaque assay.

### Mini-genome assay

HEK-293 cells (2.0×10^5^) were cultured in 24 well plates at 37°C for 1 day. After incubation, 0.5 µg of vNP-luc plasmid transfected into the cells alongside the other plasmids (0.5 µg of PB2/pCAGGS, PB1/PCAGGS, PA/pCAGGS or NP/pCAGGS, or S9A-NP/pCAGGS) using the FuGENE HD transfection reagent at 37°C for 48 h. Cells were lysed in Piccagene luciferase lysis buffer (Toyo Ink) according to the manufacturer's protocol and luciferase activity was measured with a luminometer using the Piccagene luciferase assay system (Toyo Ink).

### Purification of mRFP-Flag conjugated NP proteins

COS-7 cells were transfected with 10 µg of a pCAGGS encoding mRFP-Flag-conjugated NP110aa, NP14-110aa, R8A-NP110aa, S9A-NP110aa or mRFP-Flag alone using the FuGENE HD transfection reagent for 2 days. Cells were collected and lysed in 500 µl lysis buffer (10 mM Tris-HCl (pH 7.8), 150 mM NaCl, 1 mM EDTA and 1% NP-40) at 4°C for 1 h. After centrifuging at 20,000×*g* for 10 min, the supernatants were added to ANTI-FLAG M2 Agarose beads (Sigma) and incubated at 4°C for 1 day with gentle rotation. The agarose was washed three times with wash buffer (10 mM Tris-HCl (pH 7.4), 150 mM NaCl and 0.05% NP-40). The mRFP-Flag conjugated proteins were then eluted from the beads using the 3× Flag peptide (Sigma). The purified proteins were subjected to SDS-PAGE and Western blotting with an anti-Flag monoclonal antibody (MAb) (M2; Sigma).

### Purification of importin α proteins

GST-tagged importin α proteins were expressed and purified as described previously [19]. *Escherichia coli* expressing GST-tagged Rch1, Qip1 or NPI-1 were cultured with 1 mM IPTG at 18°C overnight. After centrifuging at 1,700×*g*, pellets were lysed in 1 ml lysis buffer containing 50 mM Tris-HCl (pH 8.0), 10% glycerol, 0.5 M NaCl, 5 mM MgCl_2_ and 0.5% CHAPS. GST-tagged proteins in the supernatant were collected by binding to glutathione-Sepharose 4B (Amersham Pharmacia Biotech). The importin α proteins bound to the GST beads were subjected to SDS-PAGE. To prepare GST-free proteins, GST-fused proteins bound to glutathione-Sepharose 4B were digested with PreScission protease (Amersham Pharmacia Biotech) as described by Kamata *et al.* [41].

### GST-pull down

Purified mRFP-Flag-conjugated NP110aa, NP14-110aa, R8A-NP110aa, and S9A-NP110aa proteins were incubated with the importin α proteins bound to GST beads at 4°C overnight. Unbound GST beads were used as a control. The beads were washed three times with wash buffer and the bound proteins were eluted by incubation with SDS-PAGE sample buffer at 100°C for 5 min. Binding of mRFP-Flag conjugated NP110aa, NP14-110aa, 8A-NP110aa or S9A-NP110aa to importin α was detected by Western blotting with anti-Flag M2 MAb.

### Standard curve for the amount of importin α proteins

To quantify the amount of importin α proteins by scanning the band image of Western blotting, different concentration of purified Rch1, Qip1 and NPI-1 proteins (0, 5, 25, 50 uM) were subjected to SDS-PAGE and western blotting. The band intensity was measured using Image J (http://rsb.info.nih.gov/ij/). The standard curve was made for concentration of purified importin α proteins and band intensity of Western blotting.

### Competitive binding assay

To analyze the binding specificity of importin α for the mRFP-Flag-conjugated NP110aa protein, 50 µM of purified Rch1, Qip1 or NPI-1 protein were added to mRFP-Flag-conjugated WT or S9A NP110aa protein bound or not bound to FLAG beads. After incubation at 4°C overnight, the beads were washed three times with wash buffer. The bound proteins were eluted with SDS-PAGE sample buffer at 100°C for 5 min. The binding of importin α to mRFP-Flag-conjugated NP110aa was detected by Western blotting with antibodies specific for Rch1 (Medical and Biological Laboratories), Qip1 (Medical and Biological Laboratories) or NPI-1 (Bio Matrix Research).

### Immunoprecipitation assay

HEK-293T cells were transfected with 10 µg of a pCAGGS encoding mRFP-Flag-conjugated WT full length NP, WT and S9A NP110aa using the FuGENE HD transfection reagent for 2 days. Proteins were purified according to “Purification of mRFP-Flag conjugated NP proteins” of Materials and Methods section. The bound proteins were eluted with SDS-PAGE sample buffer at 100°C for 5 min. The binding of importin α to mRFP-Flag-conjugated WT full length NP, WT and S9A NP110aa was detected by Western blotting with antibodies specific for Rch1, Qip1or NPI-1. The same amount of lysate was used for control.

### Silencing of Qip1

Stealth siRNA duplexes were purchased from Invitrogen and resuspended in diethyl pyrocarbonate-treated water at a final concentration of 20 µM. The sequence of the Qip1 siRNA was CAGUGAUCGAAAUCCACCAAUUGAU. Transfection of siRNA was performed using Lipofectamine RNAiMAX (Invitrogen) according to the manufacturer's protocol. Briefly, A549 and HEK-293 cells (7×10^5^ cells) were cultured in 6 well plates at 37°C for 1 day. siRNA (final concentration of 50 nM) and 5 µl of Lipofectamine were mixed with 500 µl of Opti-MEM (Invitrogen) and the mixture was incubated at room temperature for 20 min to allow the siRNA-Lipofectamine complexes to form. After replacing the medium with serum-free medium, the siRNA-Lipofectamine complexes were added to each well. Silencing of Qip1 was confirmed by Western blotting.

### Calculation of the conservation ratio

Complete sequences for influenza A virus NP were downloaded using the advanced database search at the NCBI's Influenza Virus Resource [42]. Avian and human sequences were collected using the following parameters: type A, full-length only, and whole collection date. The MAFFT (version 6) program was used to align the sequences from both groups using the FFT-NS-2 strategy [43]. Subsequently, all defective sequences were removed and then translated into amino acid sequences using GENETYX version 10 (GENETYX corporation). Finally, the obtained 6,748 human- and 3,017 avian-origin NP sequences were used to calculate the amino acid conservation ratio using the newly developed perl script program.
