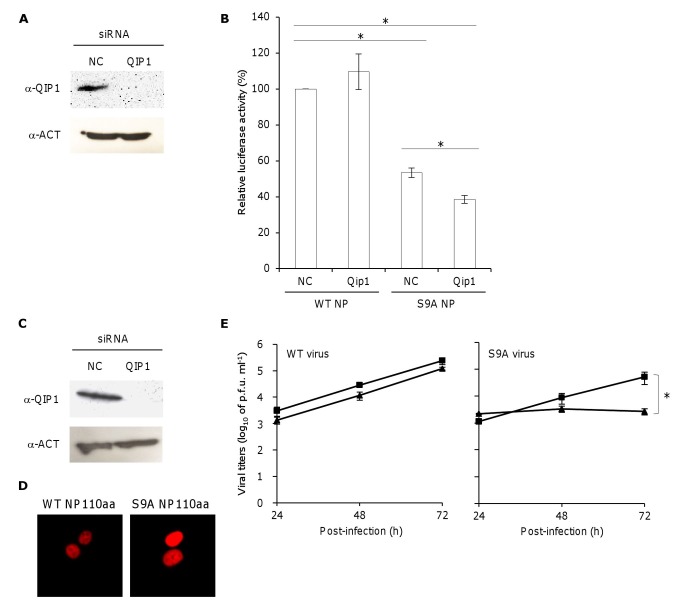# Correction: Importin α3/Qip1 Is Involved in Multiplication of Mutant Influenza Virus with Alanine Mutation at Amino Acid 9 Independently of Nuclear Transport Function

**DOI:** 10.1371/annotation/38e16278-983f-4cc0-a3b6-b16d078a5d70

**Published:** 2013-09-25

**Authors:** Yutaka Sasaki, Kyoji Hagiwara, Michinori Kakisaka, Kazunori Yamada, Tomoyuki Murakami, Yoko Aida

There are multiple errors in Figures 3B, 5B and 5E. Please find the corrected figures here:

Figure 3: 

**Figure:**
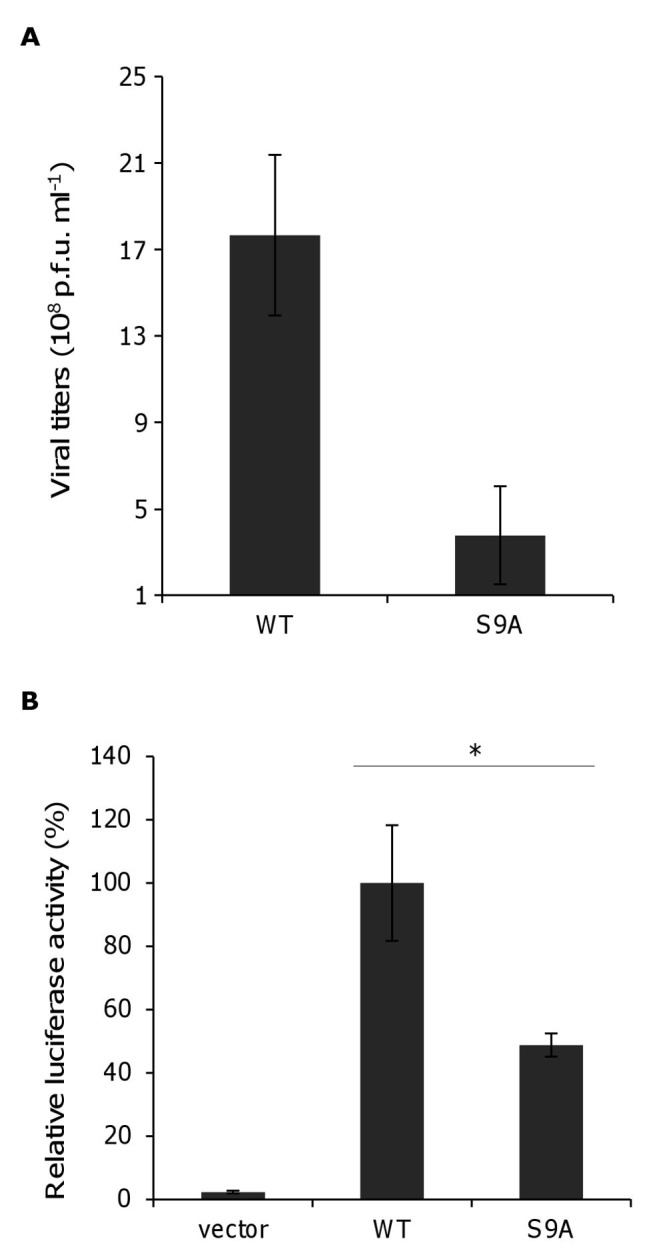


Figure 5: 

**Figure:**